# Cross-checking journalistic fact-checkers: The role of sampling and scaling in interpreting false and misleading statements

**DOI:** 10.1371/journal.pone.0289004

**Published:** 2023-07-25

**Authors:** David M. Markowitz, Timothy R. Levine, Kim B. Serota, Alivia D. Moore

**Affiliations:** 1 Department of Communication, Michigan State University, East Lansing, MI, United States of America; 2 Department of Communication Studies, University of Alabama at Birmingham, Birmingham, AL, United States of America; 3 Department of Management and Marketing, School of Business Administration, Oakland University, Oakland, MI, United States of America; 4 Department of Communication, Cornell University, Ithaca, NY, United States of America; Universiti Sains Malaysia, MALAYSIA

## Abstract

Professional fact-checkers and fact-checking organizations provide a critical public service. Skeptics of modern media, however, often question the accuracy and objectivity of fact-checkers. The current study assessed agreement among two independent fact-checkers, *The Washington Post* and PolitiFact, regarding the false and misleading statements of then President Donald J. Trump. Differences in statement selection and deceptiveness scaling were investigated. *The Washington Post* checked PolitiFact fact-checks 77.4% of the time (22.6% selection disagreement). Moderate agreement was observed for deceptiveness scaling. Nearly complete agreement was observed for bottom-line attributed veracity. Additional cross-checking with other sources (Snopes, FactCheck.org), original sources, and with fact-checking for the first 100 days of President Joe Biden’s administration were inconsistent with potential ideology effects. Our evidence suggests fact-checking is a difficult enterprise, there is considerable variability between fact-checkers in the raw number of statements that are checked, and finally, selection and scaling account for apparent discrepancies among fact-checkers.

## Introduction

Fact-checking—or the systematic assessment and publication of claims made by organizations or public figures to assess their validity [1]—is a challenging task, with the goal of helping people separate fact from fiction. Consequently, professional checkers and fact-checking organizations perform a substantial public service by identifying information that is true and false across the globe [2–5]. People, however, may question the credibility of fact-checkers for different reasons, including the perception that fact-checkers are biased, concerns around disinformation, or a general distrust in the media or institutions. A crucial question is: How do we know the fact-checkers are accurate? The answer is to check and compare the fact-checkers [6, 7].

Comparing fact-checkers raises a host of issues. The first issue involves sampling or statement selection. The sampling frame for fact-checkers is large (e.g., selecting which statements to check), especially for a public figure such as an American president. There are many more checkable statements than can practically be checked. To cross-validate one fact-checker with another, we must first determine the extent to which they check the same statements, and why. If one organization identifies more false statements than another for a certain public figure, there can be at least two plausible explanations: (a) one organization might be checking more statements, or (b) the organizations might be selecting different statements to check. If fact-checkers do not focus on the same statements, cross-checking is not viable.

A second key issue relates to a fact-checker’s philosophy on what constitutes a false and misleading statement as well as the gradations of deceptiveness. When evaluating the same statement, apparent disagreements can occur for several reasons. Fact-checkers may overlook or misinterpret evidence or apply different standards for assessing the degree of truthfulness in a statement. Messages in the wild may not be categorically true or false, and statements that are literally true can also be misleading [8–10]. When there is false content, it is typically mixed with truthful information [10]. Further still, honest messages are often “packaged”–they contain normative linguistic elements like politeness that are contextualized to make unpleasant truths palatable, thus creating a difference between acceptably honest statements and blunt truths [11]. Defining what is false and misleading is ambiguous, and organizations may differ in what counts as truthful, false, or misleading. This leads to our first research question:

RQ_1_: How do major fact-checking organizations vary with regard to triggers for uncertainty about a statement’s veracity?

Relatedly, some fact-checkers scale the nature of false and misleading statements by providing a “grade” for their deceptiveness. PolitiFact, for example, rates false statements on their Truth-O-Meter from *true* and *mostly true* to *half true* (e.g., a partially accurate statement with key omissions) to *mostly false* (e.g., a slightly truthful statement that removes critical facts), to *false* (e.g., an inaccurate statement), to *pants on fire* (e.g., an inaccurate and “ridiculous” claim) [12]. *The Washington Post* Fact Checker, in contrast, scales false statements from 1 Pinocchio (e.g., a “shading of the facts”) to 4 Pinocchios (e.g., “whoppers”). To further complicate the situation, differences in these scaling approaches may be less about definitions than scale sensitivity. In other words, one checker may be a harder grader than the other. The user takeaway from competing fact-checkers may not be the same and in the current work, we investigate the degree to which top fact-checkers are grading facts similarly.

Against this backdrop, the current paper “checked the checkers” by treating fact-checking sources like a media scholar might evaluate the accuracy of a coder in a content analysis [13], that is, by assessing fact-checkers for inter-coder agreement. We specifically compared statements by former President Donald J. Trump checked by *The Washington Post* [14] to the same statements checked by PolitiFact [15]. We also evaluated how other fact-checkers from non-journalistic outlets (i.e., Snopes, FactCheck.org) compared in their assessment of statements fact-checked. We therefore evaluated the extent to which fact-checkers are checking the same statements and if cross-validation is possible. The ability to cross-check and, by implication, the ability of fact-check readers to trust results across multiple fact-checking organizations leads to our second research question:

RQ_2_: To what degree do the scales used by different organizations yield similar interpretations of fact-checking results?

### The role of fact-checking in political journalism

Fact-gathering and fact-checking are foundational to journalism and politics [16, 17]. Journalists often advocate for and value fact-checking because it coincides with their professional values and offers a possible solution to public distrust in news and institutions [18]. Fact-checking is no longer optional for major, well-intentioned newsrooms and news organizations; it has become a central need and ethos for them in the wake of misinformation and propaganda, requiring journalists to make fast and accurate decisions about content veracity [19]. Citizens also believe fact-checking is crucial, especially for politicians [20]. Identifying the truth, and communicating it in a way that creates an informed public, are core parts of the journalism profession and are instrumental to a democracy [21]. Fact-checking is part of this process because it promotes accountability and attempts to ensure that people receive high-quality information from their leaders [22]. Additionally, to the extent that valid fact-checking is a time-consuming, effortful, and difficult task that benefits from professional training and experience, fact-checkers aid the public by making veracity assessments more accessible.

The critical issues evaluated in this work are how much two high profile fact-checkers observing U.S. politics—*The Washington Post*’s Fact Checker and PolitiFact—agree on what to fact-check and, when they do check the same facts, the degree of veracity agreement. Should two (or more) fact-checkers overwhelmingly agree on what is worth fact-checking and agree in their assessments of the factual accuracy, news consumers should have trust and confidence in a consistent set of facts from news providers to stay informed. Should there be considerable disagreement between fact-checkers on what to check, news consumers might question what they read, distrust news media, and consider fact-checkers to be partisan. They may also be left with doubts about what constitutes ground truth.

We note that prior work has taken a similar approach by evaluating statement selection from one presidency [7]. Nevertheless, there are outstanding questions to resolve. First, Lim [7] only evaluated President Trump’s statements as a presidential candidate and those fact-checked by *The Washington Post* and PolitiFact between September 2013 and November 2016. Statements during the Trump presidency were unevaluated, and it is therefore unclear how fact-checkers might have selected statements to check as President Trump took office and his deception rate increased [23]. Second, the severity or grade of a false statement (e.g., 1 Pinocchio vs. 4 Pinocchios) likely matters for how people evaluate the truth. If fact-checkers show small-to-middling agreement on fact-checking deceptiveness, this might suggest scale sensitivity discrepancies or differences in how deception is conceptualized by sources. Finally, we evaluate if fact-checking selection disagreements are moderated by the political party in office. Our work considers fact-checked statements for President Trump while in office and compared the rate of selection disagreements to the first 100 days of President Biden’s term. This helps ensure that our analysis is non-partisan and can generalize beyond a single administration.

### Fact-checking President Donald J. Trump

Honesty matters to an electorate [24, 25], and fact-checking is a key way to provide a report card for deceptive politicians. The interest in fact-checking presidents has increased over time, not only because it is seen as an innovation to journalism but also as an obligation of journalists [26]. Online access to technology and resources at scale, which began accelerating during the time of the Obama presidency, have amplified and accelerated the ability to record and verify information [6] and consequently, one of the most fact-checked public figure has been former President Trump [27, 28]. Given the sheer number of Trump’s falsehoods [23], because his approval ratings remained relatively flat as his falsehoods increased over time [29], and because his statements were consequential for the U.S. population, we evaluated how much fact-checkers agreed on their assessment of his statements. Empirically, President Trump’s statements yielded a sample size larger than any other public figure in the U.S. or elsewhere, providing a unique opportunity to evaluate fact-checkers.

Finally, we believe it is critical to evaluate statements in this manner because citizens should rely on fact-checkers to “get it right,” and report correctly and consistently on facts. If the news is not vetted properly or in a reliable manner, this can lead to the unchecked spread of fake news and disinformation. Most people are poor “fake news” detectors [30], suggesting Trump’s repeated falsehoods may have subtly undermined the public’s ability to discriminate truths from lies [31], or the public’s trust that news organizations will present facts honestly. The public expects fact-checkers and news media to keep them informed about politics, which begins with a consistent set of facts.

### Truth-default theory: Fact-checking as deception detection?

Fact-checking and deception detection are not synonymous, but the overlap is considerable. According to most definitions of deception, false statements are not lies if the sender lacks deceptive intent [32]. A politician, for example, may believe what they say, even if it is patently false. In such cases, the statement is not a lie, but it could be fact-checked as false or misleading. Further, public figures may lie about non-factual matters (e.g., opinions that are not rooted in fact). Not all false and misleading statements can be rectified by fact-checking and the intersection between what can be documented as false and what is deceptive is imperfect. Nevertheless, the overlap between deception detection and fact-checking is substantial and consequently, theories of deception detection may inform fact-checking.

Most deception detection research has investigated lie detection based on senders’ specific and observable behaviors referred to as deception cues [33, 34]. Decades of research on cue-based lie detection suggest that people are poor lie detectors (54% accuracy; [35]). In a finding called the “veracity effect,” people are consistently worse at identifying lies than truths [36]. Thus, the 54% accuracy result is correct truth-lie discrimination; but the probability of correctly identifying a lie, per se, is below 50% [35].

Truth-default theory [32, 37] provides important insights that are relevant to the current discussion, and we draw on TDT for much of our theoretical grounding. TDT is unique among theories of deception detection in its focus on communication content rather than cues, and in addition to fact-checking as a method of deception detection. Further, TDT’s concept of triggers may also be useful in understanding journalistic fact-checking. Simply stated, a trigger is any stimulus that either prompts suspicion or skepticism, or that shifts a veracity assessment from under suspicion to considered deceptive.

The core idea of TDT is that of people defaulting to the truth. This proposition is rooted in the social need for efficient and effective communication. People tend to uncritically believe communication content unless skepticism is actively triggered [37]. Consequently, the public’s vulnerability to deception is likely greater than deception detection research suggests because deception detection experiments prompt conscious truth-lie assessments that might not otherwise come to mind. Truth-default research shows, for example, that a communicator’s identity as a well-known politician, for example, is not sufficient to trigger critical considerations of veracity [38]. Outside of the laboratory, the idea that some content might be false or misleading often does not register. Fact-checking can thus serve as a trigger for the public to assess veracity and honesty [32]. Interestingly, the trigger construct may also apply to fact checker’s selections of what to fact check. Differential trigger sensitivity may offer a useful theoretical frame for understanding differences in selection criteria and choices.

Truth-default theory prioritizes checking facts over behavioral cues and demeanor [32, 37]. The theory values journalistic fact-checking because (a) checking facts is theoretically specified as the most preferred and efficacious method of deception detection, and (b) due to the truth-default, the public is unlikely to critically assess content in the absence of dedicated fact-checkers. People tend to believe that others are honest, most of the time. Evidence of the efficacy in fact-checking by identifying false and misleading statements would be consistent with the theory; robust inaccuracies would undercut a major assumption of the theory.

Presently, most fact-checking is a human activity. Therefore, issues of how a fact-check is triggered, the ways in which it yields information to confirm or disconfirm the veracity of a statement, and its ability to be shared with many people rather than informing the judgment of a single individual are important consequences to applying the TDT propositions. Importantly, TDT states (regardless of who is doing the detecting), deception is most accurately detected, “by comparison of the contextualized communication content to some external evidence or preexisting knowledge” [37]. As this seems to be the essence of fact-checking, we therefore raise the third research question:

RQ_3_: To what degree does truth-default theory apply to journalistic fact-checking?

### The current study

In the U.S., two of the most prominent media-based political fact-checkers are *The Washington Post* Fact Checker and PolitiFact. The present work draws on the perspective of truth-default theory to evaluate how much these fact-checkers agree in their assessment of facts. Here, we focus on how much overlap exists among statements that are checked by leading fact-checkers (i.e., the influence of human abilities). We also evaluate the strength of the association between scaling across *The Washington Post* Fact Checker and PolitiFact to assess if they frequently give the same or similar deceptiveness ratings. Finally, to broaden the scope of our investigation, we evaluated how often two non-journalistic sources (i.e., Snopes, FactCheck.org) fact-checked the same statements as PolitiFact.

## Method

### Data collection

#### *The Washington Post* Fact Checker

The Fact Checker is an ongoing feature that examines the truth of statements by political figures, ranging from local to international, and relies on reader input for topics to fact check. The database of Donald Trump’s false and misleading statements from *The Washington Post*’s Fact Checker (*N* = 30,573 statements) was obtained from the database creators [23]. The database is a very large, although not complete, collection of statements. This project evolved from a post-election examination of Trump’s public statements, to a comprehensive look at his first 100 days, to a daily tally until the end of his term. While *The Washington Post* generally relies on reader suggestions and tips regarding erroneous claims, the Fact Checker attempted to evaluate all topics and claims. Metadata in this archive included each false and misleading statement, the statement date and analysis, and a 4-point deceptiveness scaling (e.g., 1 to 4 Pinocchios). Importantly, only statements rated 2 to 4 Pinocchios were provided by *The Washington Post*. Statements judged to be true are not included (i.e., *The Washington Post* only reported on false or misleading statements), and Bottomless Pinocchios (e.g., repeated false or misleading statements) were not considered for this analysis to avoid redundancy. Overall, *The Washington Post* has the most extensive catalog of the former president’s deceptive claims.

#### PolitiFact and other fact-checkers

We collected fact-checked statements from PolitiFact (*N* = 938) by locating Trump’s scorecard and only considering statements made during his term in office. Each statement contained a range of data, including the 6-point deceptiveness scaling (e.g., *true*, *mostly true*, *half true*, *mostly false*, *false*, *pants on fire*), the date of the statement, when it was fact-checked, and who fact-checked the statement. The large Fact Checker database provided the impetus for this analysis. But we used the PolitiFact data as the focal dataset because it contained true and false veracity ratings; *The Washington Post* has only false statements. Other fact-checkers are compared to PolitiFact.

Unlike *The Washington Post* project, which monitored all of Trump’s public statements and catalogued those which are unambiguously false, PolitiFact applied its usual selection process to Trump’s statements. Typically, PolitiFact performs preliminary reviews of news stories, press releases, political speeches and advertising, campaign websites, and social media [12]. In addition, it accepts reader suggestions. PolitiFact then determines what to fact check using several criteria: Does it have a verifiable fact (they do not fact-check opinions)? Does it leave a misleading impression? Is it a significant statement (e.g., not an obvious misstatement)? And, is it likely to be passed on and repeated? Consequently, PolitiFact puts less emphasis on the quantity of Trump’s deceptive statements and misinformation. Instead, its subjective focus is on those statements that are likely to be newsworthy and socially impactful.

We took a layered approach to evaluate how often statements fact-checked by other popular sources were cross-checked with PolitiFact. First, we evaluated only statements from *The Washington Post* that were also examined and classified by PolitiFact in the *half true*, *mostly false*, *false*, and *pants on fire* categories, since these statements were judged to contain explicit elements of deception (final sample, *n* = 513 statements during Trump’s presidency). We could not cross-check *true* or *mostly true* statements by PolitiFact (*n* = 64) because these statement types did not exist in *The Washington Post* database. To assess the possibility that the 64 *true* or *mostly true* PolitiFact statements might be differentially classified as false by *The Washington Post*, we also conducted a verbatim match (not requiring coding) against the 2 to 4 Pinocchio statements.

To further address this limitation, in a second analysis we selected a random sample of 100 statements across all six major PolitiFact deceptiveness categories (*true*, *mostly true*, *half true*, *mostly false*, *false*, *pants on fire*) and evaluated the selection correspondence with Snopes and FactCheck.org. These two sources are among the top non-journalistic fact-checkers and together, we evaluated how often PolitiFact statements are checked or unchecked by all three sources (*The Washington Post*, Snopes, FactCheck.org). While the criteria for moving forward with a fact-check are similar to those of PolitiFact, both Snopes and FactCheck.org have different approaches to the initial selection. Snopes covers a broader range of topics, not limited to politics, which are determined primarily by the strength of reader interest. FactCheck.org limits itself with regard to who gets checked (President Trump was within their focus), seeks claims of national significance, and typically ceases checking when a claim appears to be true. While claims by Trump have been fact-checked by all four organizations, the breadth of statements checked varies considerably and is determined by the objectives of the organization.

### Coding procedure

#### Cross-checking PolitiFact and *The Washington Post*

Two coders, the first and fourth authors, independently searched each PolitiFact statement to identify if they also appeared in *The Washington Post*’s list (Since the number of statements in *The Washington Post* database was substantially larger than the number of statements in the PolitiFact database, we directed our search by identifying if PolitiFact statements were also found in *The Washington Post*, but not the reverse). Each coder relied on three criteria, in combination, to determine an appropriate match was made between sources: (1) a verbatim phrase correspondence (about two to five words), (2) date proximity (e.g., if the statements occurred on the exact date or were within three days of each other), and (3) topic similarity (e.g., the topic of each statement must be equivalent). In a few cases, verbatim matches were impossible due to subtle transcription differences between sources or if a single word indicated a false statement. For example, consider the PolitiFact fact-check, “Video shows California election workers ‘cheating’ by collecting ballots from drop box on Nov. 4,” stated on November 11, 2020. Except for one word (“cheating”), the statement would not allow for a verbatim match because the rest of the statement mostly provided background or context to the fact-check. The statement still made a general reference to possible election cheating, however. In such cases, we ensured that the same topic was discussed across fact-checks and the date proximity of both statements were within three days. Variations in dates were typically due to factors such as the release date for transcripts or whether the source was primary (e.g., obtained from a tweet or live speech) or secondary (e.g., media reporting).

Coders made a binary judgment (1 = yes, 0 = no) determining if a PolitiFact statement also appeared in *The Washington Post*’s list. Raw agreement was substantial (476/513; 92.8%), and intercoder reliability, which adjusts for guess-rate or judgments being due to chance, was also strong (Krippendorff’s α = 0.797, bootstrapped 95% CI [.731, .863] using 5,000 replicates; see S1 File for more details). Coders resolved cross-checking discrepancies after discussion and re-performing the fact-checks together to ensure the right cross-check occurred. Please see the S1 File for examples of five representative and successful cross-checks across *The Washington Post* and PolitiFact. Note, since this coding procedure was a simple cross-checking task and did not involve thematic interpretation akin to qualitative coding, special coding software was not required. Please see the S1 File for a description of how deceptiveness ratings were aligned across sources.

#### Cross-checking PolitiFact with Snopes and FactCheck.org

The first and second authors independently coded a random selection of 100 PolitiFact fact-checks and evaluated if the statement was also checked by Snopes or FactCheck.org. Coders entered a statement or keywords from the statement into the website’s search bar and read relevant articles on each site to identify if a fact-check about the exact statement’s topic or issue was made. Statements were placed into one of three categories: (1) Checked, and source (Snopes or FactCheck.org) agrees with PolitiFact on veracity, (2) Not checked by source, or (3) Checked, but source disagrees with PolitiFact on veracity. This procedure occurred for Snopes and FactCheck.org, independently.

Initial coding of these statements reached an acceptable percent agreement for Snopes (94% agreement) and FactCheck.org (82% agreement). Note, we did not compute traditional intercoder reliability statistics because the number of fact-check “hits” (e.g., the number of times that a PolitiFact statement was also found on Snopes and FactCheck.org) was low and the total number of reviewed statements was small as well, which would bias reliability calculations. Discrepancies were resolved after discussion and coders re-performing the fact-checks together.

Data are available from the authors or *The Washington Post* by contacting Glenn Kessler, maintainer of the Fact Checker (Glenn.Kessler@washpost.com).

## Results

### Cross-checking selection agreement

Our cross-checking revealed 22.6% of statements fact-checked by PolitiFact did not appear in *The Washington Post* database (116/513) (An exploratory thematic analysis using the Meaning Extraction Method [39, 40] for the statements PolitiFact checked but *The Washington Post* did not is available in the S1 File). As the top panel of Table 1 suggests, a range of 20–35% of the statements across the four deceptiveness rating categories used by PolitiFact were not checked by *The Washington Post* or were not included in the database of 2–4 Pinocchio statements. The verbatim check for *true* and *mostly true* statements found that only 1 out of 64 statements (1.6%) was given a false and misleading rating and included in *The Washington Post* selection, when PolitiFact tagged it as *mostly true*. This discrepancy is investigated further in the S1 File.

**Table 1:** Summary of cross-checking results across analyses for President Trump data.

Panel 1	PolitiFact Rating					
Source	--	--	Half true	Mostly false	False	Pants on fire
PolitiFact			68	122	224	99
*The Washington Post*			44	98	177	79
Cross-check selection agreement (%)			64.7	80.3	79.0	79.8
Cross-check selection disagreement (%)			35.3	19.7	21.0	20.2
Panel 2	PolitiFact Rating					
Source	True	Mostly true	Half true	Mostly false	False	Pants on fire
PolitiFact	5	11	5	22	33	22
Snopes	0	0	1	2	3	2
FactCheck.org	1	2	1	4	9	4
Cross-check selection agreement (%)	20.0	18.2	40.0	27.3	36.4	27.3
Cross-check selection disagreement (%)	80.0	81.8	60.0	72.7	63.6	72.7

*Note*. The denominator in each cross-check agreement calculation is the PolitiFact value. For example, 64.7% of PolitiFact *half true* statements were also observed in *The Washington Post* database. For the cross-checking with Snopes and PolitiFact, two cases had the label as “full flop,” which reflects a change in position on an issue, but not necessarily deception. Therefore, cases representing this category were excluded and the number of fact-checked statements for PolitiFact sums to 98, not 100. The cross-check agreement calculation in the bottom panel represents the percent of overlap with PolitiFact by at least one of the two sources (Snopes or FactCheck.org). Here, Snopes and FactCheck.org results were summed in the numerator and PolitiFact results were the denominator.

Our general pattern of results replicated prior work well [7]. We therefore attempted to confirm that the fact-checkers are consistent in their identification of false statements in other ways. Specifically, to provide an independent assessment of these data and further understand the difficulties involved with fact-checking, all four authors independently fact-checked a random selection of 10 statements previously fact-checked by both organizations. Each coder searched online sources for a claim’s veracity without using *The Washington Post* Fact Checker, PolitiFact, or other published fact-checkers as sources. Coders made *true*, *false*, or *uncertain* ratings based on such evidence and coders also indicated the sources they used for veracity determinations. On 8 of the 10 statements, coders reached 100% agreement. The remaining 2 statements reached majority agreement (75%). In both cases, the fourth coder believed the statement was false, but felt the evidence was sufficiently ambiguous that a “false” rating could not be assigned. These data demonstrate that fact-checking is not easy, even for trained coders using clear guidelines. Poor grammar, conflating of ideas, obfuscation (intentional or not), conflicting source data for claims, and subjective interpretation of source data all contribute to the difficulty of ascertaining the veracity of a claim. Despite these challenges, our independent fact-check of the previously fact-checked statements validated the published results and therefore serves as an important robustness check.

#### Beyond the Trump presidency

One concern with our analysis relates to the perceived liberal political bias that fact-checking organizations might have against a Republican president such as Donald Trump. To address this issue, all available fact-checks during President Biden’s first 100 days were extracted from *The Washington Post*’s Fact Checker (*n* = 78). PolitiFact statements in the *half true*, *mostly false*, *false*, and *pants on fire* categories (*n* = 11) were identified and an additional cross-checking process occurred. Only one coder (the lead author) reviewed these statements because the number of cross-checks was small.

The data revealed 6 out of 11 statements (54.5%) from the PolitiFact database were also observed in *The Washington Post* Fact Checker’s list and the remainder (5/11) were not cross-checked. Note, this does not suggest one source rated them as *true* and the other did not, but instead, the statements did not meet *The Washington Post*’s criteria for fact-checking or were missed. Consistent with the cross-checks for President Trump, there is a nontrivial degree of divergence across fact-checkers in what to check. We interpret these data with caution, however, since the number of fact-checked statements for Trump during his first 100 days [41], was substantially larger than for Biden. The same selection procedure and fact-checking team were used for the first 100 days of Trump and Biden.

### Cross-checking agreement with Snopes and FactCheck.org

We collected a random sample of 100 statements fact-checked by PolitiFact and identified if they were also checked by Snopes and FactCheck.org. This analysis helped to indicate if a subtle, yet unknown fact-checking bias existed for *The Washington Post* or if a similar rate of selection agreement/disagreement existed for other sources as well.

Only eight PolitiFact statements were also checked by Snopes and 21 PolitiFact statements were also checked by FactCheck.org. No disagreements in veracity were observed and all statements fact-checked by Snopes were also checked by FactCheck.org. The bottom panel of Table 1 reveals most statements checked by PolitiFact were not checked by Snopes or FactCheck.org. At most, 40% of statements from Snopes or FactCheck.org were also checked by PolitiFact. Taken together, the evidence collectively suggests more correspondence in statement selection between *The Washington Post* and PolitiFact compared to other sources and PolitiFact (All 8 PolitiFact statements checked by Snopes were also checked by *The Washington Post* and two-thirds of the statements (14/21) also checked by FactCheck.org were checked by *The Washington Post*). The data further suggest that discrepancies are evident in what is fact-checked, but not in the bottom-line determinations of veracity (i.e., was the statement true or false?). When different fact-checkers check the same statement, they invariably agreed on the substance of the check.

### Deceptiveness scaling

We calculated a bivariate correlation between deceptiveness ratings for any statement with a Pinocchio rating from *The Washington Post*’s Fact Checker and a Truth-O-Meter rating from PolitiFact. The relationship between these deceptiveness ratings was positive and statistically significant, *r*(107) = .451, *p* < .001. A non-parametric bivariate correlation was also consistent in magnitude and operated in the same direction, ρ = .419, *p* < .001.

It is important to note that PolitiFact defines *false* as “The statement is not accurate” and *pants on fire* as “The statement is not accurate and makes a ridiculous claim.” Therefore, *pants on fire* is the scale equivalent of a *false* statement; the ridiculous nature of a claim does not alter the “falseness” of it, but instead adds a qualifier about how the claim is made. Converting values of the *pants on fire* category to *false* produced a similar result, *r*(107) = .447, *p* < .001.

**Fig 1:**
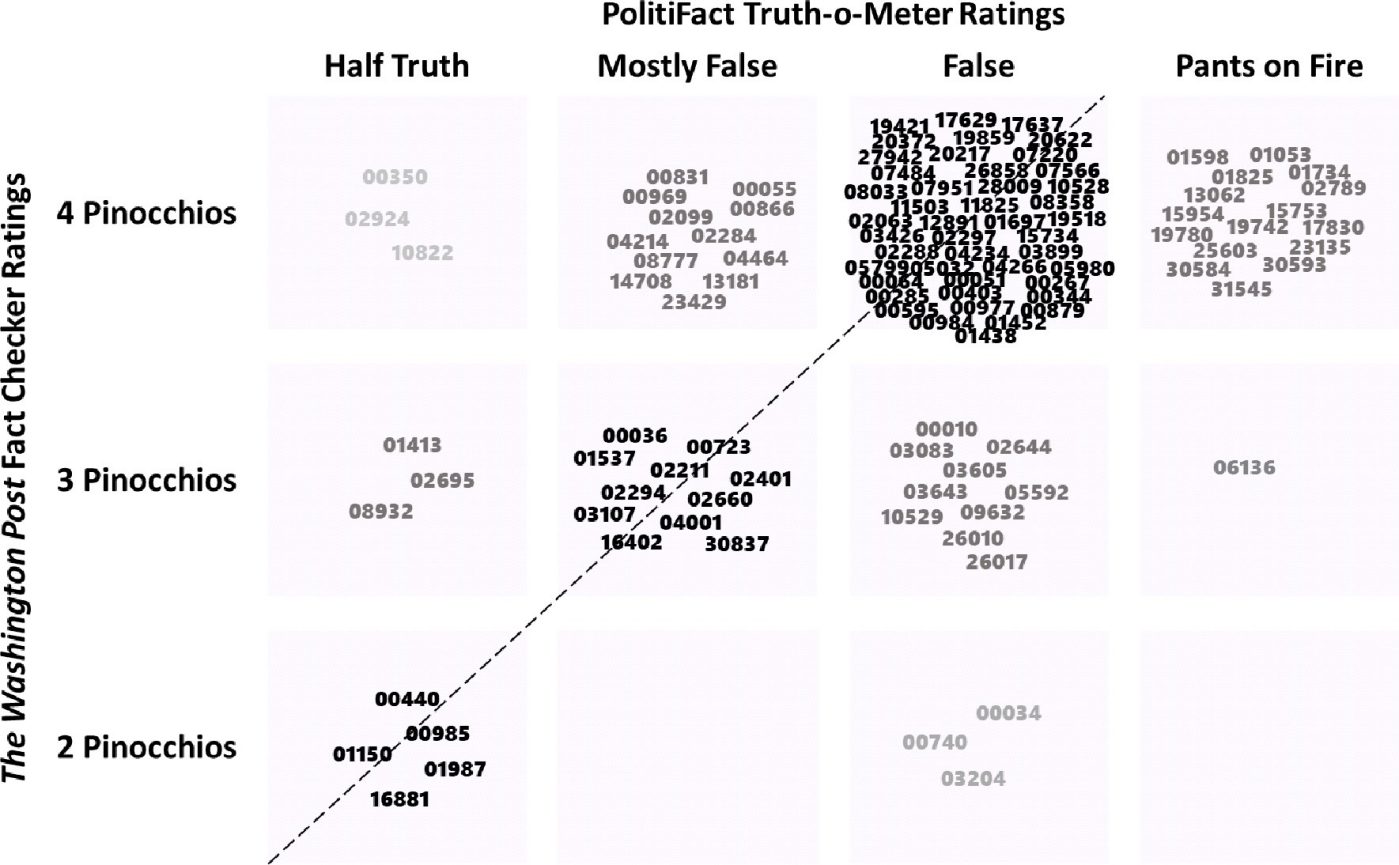
The relationship between Pinocchios from *The Washington Post* and Truth-O-Meter ratings from PolitiFact. *Note*: Numbers correspond with *The Washington Post*’s unique identifiers and are broadly arranged within cells for interpretability.

Deceptiveness agreements systematically decrease from *pants on fire* distinctions to *half truths* (Fig 1). *Half truths* have a seemingly random distribution of Pinocchios and there is near perfect agreement for *pants on fire* statements. This suggests that there is moderate deceptiveness agreement across *The Washington Post* Fact Checker’s Pinocchios and the Truth-O-Meter from PolitiFact. Correlations show general agreement but blurring of adjacent categories is also common. Most discrepancies were only off by one category, and there were only 6 instances of being off by two category discrepancies. Major off-diagonal disagreements were largely exaggerations by President Trump that *The Washington Post* considered egregious and PolitiFact believed they had some validity. For example, three examples in the top left corner of Fig 1 related to exaggerations by President Trump (e.g., “I’ve won awards on environmental protection;” 4 Pinocchio’s), but were half-truths via PolitiFact (he received a personal award for donating land in New York State after not building a golf course there).

## Discussion

This paper focused on how much two prominent fact-checking sources, *The Washington Post*’s Fact Checker and PolitiFact, agreed on the statements to fact-check and the strength of their deceptiveness ratings. The study examined presidential fact-checks, and the data suggest some divergence in what to check. Deceptiveness ratings were moderately correlated for *The Washington Post* and PolitiFact, suggesting general agreement on the presence of falsity but indicating variability in severity assessments of false or misleading statements.

Our work is among the first studies to compare leading fact-checkers across two presidents and multiple sources. As described, there are two potential reasons for the fact-checking disagreements beyond substantive errors. RQ_1_ addresses the first of these by asking how major fact-checking organizations vary regarding the fact-checking triggers. We observed that the sampling frame for each source can be different, which therefore leads to disagreements in whether statements are checked. PolitiFact indicates they check statements based on five criteria: (1) Is the statement rooted in a fact that is verifiable? (2) Does the statement seem misleading or sound wrong? (3) Is the statement significant? (4) Is the statement likely to be passed on and repeated by others? (5) Would a typical person hear or read the statement and wonder: Is that true? *The Washington Post*’s Fact Checker, on the other hand, evaluates “the statements of political figures regarding issues of great importance, be they national, international or local” [42]. According to the Fact Checker, inquiries are often provided by readers and then *The Washington Post*’s staff evaluates statements for the truth. From these descriptions, discrepancies in the sampling frame might be anticipated. PolitiFact employs a stricter set of criteria to begin the fact-check process and seemingly does not solicit input from readers. *The Washington Post*’s Fact Checker casts a wider net on the statements to fact-check. This was especially the case for President Trump as they set out to do a comprehensive review of his statements over time. These differences are noticeable and appear to affect the data.

A second reason for the fact-checking disagreements might be related to each source’s definition of deception. RQ_2_ asked whether the scales used by different organizations yield similar interpretations of the fact-checking results. In our review of *The Washington Post* and PolitiFact methods, we could not find a clear conceptual definition of deception as stated by the fact-checkers. However, each source’s operational definitions, in the form of deceptiveness ratings, offer some perspective into such conceptualizations. PolitiFact’s false statements range from omissions to ridiculous claims, while *The Washington Post*’s Fact Checker rates statements from “omissions and exaggerations, but no outright falsehoods” (1 Pinocchio) to “whoppers,” (4 Pinocchios) or outright ridiculous claims [42]. These conceptualizations appear reasonably consistent. Our correlation between deceptiveness ratings was moderate, however, suggesting the interpretation of what constitutes a false or misleading statement is crucial to fact-checking. Despite goals of objectivity in fact-checking and journalism, subjectivity is still a part of this enterprise, and that subjectivity appears when judgments are made about the degree of a statement’s deceptiveness. There seems to be a fairly high level of agreement when a statement is false, but the degree of falseness appears to vary. This variation is not egregious, but it leaves open the question of whether the consumers of different fact-checking results see these variations as trivial or substantive.

Taken together, our analyses lead to four top-line conclusions. First, there is considerable variability between political fact-checkers in the raw numbers of statements that are checked. This sheer volume difference is indicative of different goals by different fact-checkers. The implication is that it may not be possible to cross-check most fact-checks with other fact-checkers. Second, there is a moderate correlation in scaling. Even though most of the differences are small, such differences can create the appearance of disagreements. Thus, both selection and, to a lesser extent, scaling account for apparent discrepancies among fact-checkers. Third, bottom-line determinations of statement veracity (e.g., did the checkers agree on true-false judgments among communally fact-checked statements?) align across fact-checkers. When we independently checked statements, and when different fact-checkers independently checked the same statements, there was near consensus about whether the statement was true or false. While the data do not allow for a complete veracity match, across all of our comparisons, we found only one instance (out of 64 statements) where one checker assessed a statement as true and another assessed the same statement as potentially false. Disagreements were in degrees or confidence, not in kind. This leads to our fourth and final conclusion: political fact-checking is difficult, nuanced, and yet remarkably consistent across checkers once they agree on what to check. Fact-checking exhibits bottom-line validity and merits confidence.

### Implications for political fact-checking, news consumers, and deception theory

The current results have several implications for political fact-checkers, media scholars, and news consumers. Although understandable, differences in statement selection are substantial.

Journalists and political fact-checkers should be aware of uniquely high-frequency deceivers (prolific liars, or those who lie prolifically; [43–47]) and when they might be more likely to lie (e.g., as suggested by TDT, when topics are consequential or when the truth is a problem; [32]). For example, during his final year as president and as the 2020 U.S. presidential election approached, President Trump’s lying rate increased dramatically compared to other years [23]. Thus, fact-check frequency should increase proportionally. Yet, fact-checkers have finite resources and they must be selective. Newsrooms and political fact-checkers should create processes to be systematic and comprehensive with their cataloguing of deception, while adjusting to variable base-rates of lying. Relatedly, the fact-checkers have a responsibility to be unbiased and balanced in their approach. Politicians and political candidates may be unequal in their frequency of deceptive messaging, but the fact-checking procedures should reveal that imbalance rather than focusing on politicians who create an *a priori* expectation of dishonesty. Finally, while selection variation may be clear to researchers, the implications may not be so clear to those using these fact-checking resources. Fact-checkers need to communicate these differences with greater precision and clarity to the public.

This investigation provides an understanding of fact-checkers’ and political fact-checking blind spots. When fact-checkers disagree on what to fact-check, and their disagreements are prevalent, news consumers might doubt what they read and how much they trust a source. The implications of fact-checking disagreements are exacerbated when they become aggregated in news stories, read by a wide circulation on social media, or when a concerned citizen cannot find the same statement fact-checked by another source. News consumers should not have to doubt or fact-check the fact-checkers. Perhaps having a consistent set of standards in journalistic fact-checking (e.g., definitions of deception, deceptiveness rating systems that are consistent across sources) and removing ad-hoc policies at the source level will alleviate public concerns about fact assessments. More widely promoting and creating public awareness of fact-checking standards might also be beneficial (https://www.poynter.org/ifcn/).

Finally, in response to RQ_3_ about the degree to which TDT applies to journalistic fact-checking, it is also important to describe how our results inform truth-default theory [32, 37]. Our evidence suggests bottom-line judgments of statement veracity were almost perfectly aligned for professional fact-checkers. Among those checked by PolitiFact, there were broad similarities and modest differences across scaling compared to those checked by *The Washington Post*. Therefore, fact-checkers agree on veracity when they both check a statement, but their selection and scaling can be misaligned. These results inform truth-default theory because the theory champions fact-checking (labeled in the theory as correspondence information) as one of the most preferred methods of lie detection. Our results show that fact-checking appears valid, and the theory can be applied to non-interactive (e.g., not interpersonal) settings of consequence (e.g., political and journalistic fact-checking). The process of detecting deception is not limited to one individual confirming the veracity of another. The triggers for suspicion can arise from many sources, and while the fact-checking organization functions in ways that are analogous to an individual’s efforts to confirm or disconfirm a message, the results may be widely circulated to a mass audience of news consumers.

According to truth-default theory, most people presume a message is honest independent of its actual veracity [32, 37]. Therefore, if false or misleading statements are not fact-checked (or not agreed upon to be fact-checked by multiple sources), their ability to mislead the public goes unchallenged. The truth-default remains until a trigger exists. Fact-checking can serve as a trigger or be the response to a trigger. Nevertheless, absent public confidence in fact-checkers, the utility of the triggering function is undercut.

Our test of TDT in the domain of fact-checking has implications beyond U.S. fact-checking sources like *The Washington Post* Fact Checker and PolitiFact because the theory is pan-cultural [32, 37]. As prior work suggests, the theory’s “core presumptions concern human nature and are not tied to any particular culture, religion, government, or social structure” [44], suggesting that its principles, propositions, and foundations can extend to fact-checking in other regions of the world. Fact-checking efforts are global due to the pervasive and consequential nature of disinformation [2–5] and therefore, it may be helpful for future work to compare how fact-checking approaches can inform each other in a pursuit of best practices, identifying elusive perpetrators of false information, and understanding cultural differences related to the dissemination and detection of such false information.

### Lessons from political fact-checking

We learned that political fact-checking is difficult, and fact-checkers face nontrivial challenges as they try to evaluate the truth. In some cases, as our analysis involving Snopes and FactCheck.org revealed, only one organization will fact-check a claim. People often want information fast, though fact-checking requires time and thorough investigation. Even then, fact-checkers are susceptible to human error and human biases that might affect their judgment. We advocate for reliability estimates in fact-checking and cross-checking sources to ensure consistent and credible news. Two of the coders in the PolitiFact, Snopes, and FactCheck.org cross-check also noticed that FactCheck.org’s search algorithm was enhanced by Google. Therefore, if sorted by “Relevance,” coders or fact-checkers might receive slightly different search results on FactCheck.org because of a person’s unique Google history and what Google assumes the searcher would be interested in. Algorithmic bias in fact-checking and citizens’ experiences should be acknowledged.

### Limitations and future directions

Our investigations purposefully chose premier fact-checkers across analyses, but there are others that would be worthy of evaluation in future work. We selected *The Washington Post*’s Fact Checker, PolitiFact, and others because of their popularity and deceptiveness scaling, but more sources, including those outside the U.S. could be assessed as well (e.g., CNN, USA Today, Agence France-Presse, and other members of the International Fact-Checking Network). A cross-cultural analysis of fact-checking might be helpful to illuminate cultural or norms-based differences in what people decide to fact-check and how veracity might be scored differently. However, this may require new methods as the opportunity for direct comparison of checked facts is limited. We also evaluated two presidents in this paper. Therefore, it will be important to evaluate how checkers might continue to disagree on facts from other political figures.

Although the results of this work described disagreement between two main fact-checkers, it is unclear why these patterns emerged. We offered two possible explanations based on methodological and theory-based evidence, though future work would benefit from interviewing fact-checkers to understand their process and decisions on what to check in difficult cases [48]. Pairing qualitative evidence about editorial decision-making and fact-checking principles with quantitative work would make for a more holistic evaluation of how much fact-checking disagreements exist and why they might exist.

In addition, we only evaluated false statements when comparing PolitiFact and *The Washington Post*; comparing true and false statements required additional fact-checking with Snopes and FactCheck.org. Therefore, a limitation of some analyses is that we cannot evaluate potential disagreements on truths, which are also critical to fact-checking. Nonetheless, the data suggest that fact-check results are mostly accurate. It is also important to note that while coders made independent cross-checks and were reliable in the paper, future work may consider additional approaches to confirm objectivity. For example, it is unclear if artificial intelligence (e.g., ChatGPT) can perform cross-checks, but this may be an important step to consider.

Finally, our research focused on the actions of the fact-checkers. When assessing the outcomes of fact-checking, it would be useful to obtain feedback from the consumers of fact-checking news. If a news consumer observed that a fact-checker had an incorrect fact-check, does this impact the source’s credibility and if so, what can be done to repair it? While our professional judgments resulted in a high level of agreement, news consumers—who may be untrained and whose judgments may be constrained by past exposure to the candidates, biased by repetitive exposure to the messages being checked, or influenced by the values of others with whom they interact—may not interpret fact-checking results with the same clarity or be universally forgiving if a mistakenly incorrect fact-check occurs. This suggests that the results not only need to be accurate, but they need to be credible.

## Supporting information

S1 File (DOCX)
