# Supplementary material for: Cross-checking journalistic fact-checkers: The role of sampling and scaling in interpreting false and misleading statements
Source: PLoS One. 2023 Jul 25;18(7):e0289004. doi: 10.1371/journal.pone.0289004 (PMC10368232; doi:10.1371/journal.pone.0289004)
Supplement: S1 File — (DOCX) [file pone.0289004.s001.docx]

Table of Contents

[Cross-Checking Examples 2](#_Toc137114433)

[Expanded Reliability Commentary 3](#_Toc137114434)

[Expanded Deceptiveness Ratings Across Sources 5](#_Toc137114435)

[Exploratory Thematic Analysis of Unchecked Statements 6](#_Toc137114436)

[Expanded Investigation into PolitiFact and *The Washington Post* Discrepancy 8](#_Toc137114437)

[References 9](#_Toc137114438)

# **Cross-Checking Examples**

| PolitiFact | Date | Deceptiveness |
| --- | --- | --- |
| 1. "We've made other countries rich while the wealth ... of our country has dissipated over the horizon." | 1/20/17 | Mostly false |
| 1. "The media ... sort of made it sound like I had a feud with the intelligence community." | 1/21/17 | False |
| 1. Says ICE and border patrol officers "unanimously endorsed me for president." | 1/25/17 | Mostly false |
| 1. "Here in Philadelphia murder has been steady — I mean — just terribly increasing." | 1/26/17 | False |
| 1. "If you were a Muslim, you could come in, if you were a Christian, it was impossible." | 1/27/17 | False |
|  |  |  |
|  |  |  |
| The Washington Post | Date | Pinocchios |
| 1. "[We've] spent trillions and trillions of dollars overseas while America's infrastructure has fallen into disrepair and decay. We've made other countries rich, while the wealth, strength and confidence of our country has dissipated over the horizon." | 1/20/17 | -- |
| 1. "I have a running war with the media. They are among the most dishonest human beings on Earth. And they sort of made it sound like I had a feud with the intelligence community." | 1/21/17 | 3 |
| 1. "Before we go any further, I want to recognize the ICE [Immigration and Customs Enforcement] and Border Patrol officers in this room today and to honor their service and not just because they unanimously endorsed me for president." | 1/25/17 | -- |
|  |  |  |
| 1. "Here in Philadelphia, the murder rate has been steady — I mean just terribly increasing." | 1/26/17 | -- |
| 1. "They've been horribly treated. Do you know if you were a Christian in Syria, it was impossible, at least very tough, to get into the United States? If you were a Muslim, you could come in, but if you were a Christian, it was almost impossible, and the reason that was so unfair, everybody was persecuted in all fairness, but they were chopping off the heads of everybody but more so the Christians. And I thought it was very, very unfair." | 1/27/17 | 2 |

# **Expanded Reliability Commentary**


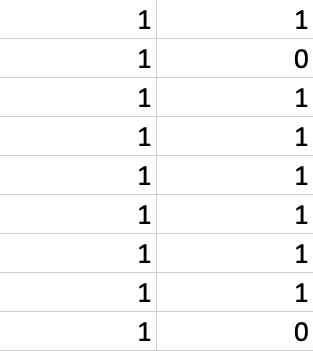
**
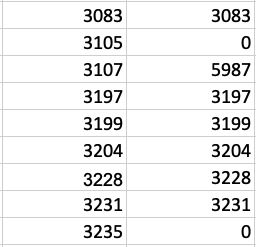
**Coding was performed to identify if accurate cross-checks occurred between judges. First, two coders independently placed unique identifiers from *The Washington Post* in separate columns of a spreadsheet if they found a cross-check correspondence between *The Washington Post* Fact Checker and PolitiFact. These identifiers were numeric. In cases where one coder believed that there was no cross-checked fact-check between sources, they kept this cell blank, and this was eventually replaced with the number “0” for reliability calculations. In practice, the combined code-sheet looked like the following example (left panel):

In the left panel, numbers that were identical across columns (e.g., 3083, 3197, etc.) represented cross-checked matches, meaning that a cross-check occurred, and the same fact-checked statement was found in both *The Washington Post* and PolitiFact databases by both coders. In row 2, however, the first coder (column 1) found the statement in question in *The Washington Post's* list under #3105, but the second coder (column 2) did not. In row 3, the first coder found the statement in question in *The Washington Post's* list under #3107, but the second coder found the statement in question in *The Washington Post's* list under #5987.

To calculate reliability, numeric responses were binned into two categories (right panel in the above figure): if a fact-check could be cross-checked (coded as 1) or not (coded as 0). Any non-zero response (e.g., 3083, 3105) was coded as a successful cross-check (coded as 1). This coding process led to strong reliability (Krippendorff’s α = 0.797). However, it was also important for coders to ensure that the *correct* cross-check was made (like row 3 in the left panel above, where the coders both agreed that a cross-check could occur, but they disagreed on the statement leading to this judgment). Coders then resolved discrepancies (58/513) between possible cross-checking differences (e.g., if a cross-check was not made, or if a different cross-check was made).

# **Expanded Deceptiveness Ratings Across Sources**

| Label | Source | Numerical value |
| --- | --- | --- |
| Half-true | PolitiFact | 3 |
| Mostly false | PolitiFact | 4 |
| False | PolitiFact | 5 |
| Pants on fire | PolitiFact | 6 |
| 2 Pinocchios | The Washington Post | 2 |
| 3 Pinocchios | The Washington Post | 3 |
| 4 Pinocchios | The Washington Post | 4 |

*Note*. The fact checking labels constitute ordinal scales indicating checked statements’ deviation from the truth. For our analysis we have assumed that the values to be equal interval. The use of Pearson (parametric) and Spearman (nonparametric) correlations with these data produce similar results. The actual values used are irrelevant except to the extent they preserved the scale order. We have retained the values assigned by the fact-checking services.

# **Exploratory Thematic Analysis of Unchecked Statements**

Are there common topics among the statements that PolitiFact fact-checked but *The* *Washington Post* did not? To assess the thematic content of such statements, we submitted the 116 selection disagreements to the Meaning Extraction Method (Chung & Pennebaker, 2008; Markowitz, 2021), an exploratory automated text analysis approach that removes function words (e.g., articles, prepositions, pronouns) and low base-rate words from a collection of texts, but retains content (e.g., nouns, verbs). Content words are then used in a Principal Component Analysis (PCA) to evaluate how they cluster statistically and form themes. Words were retained in this analysis if they appeared in at least 5% of the disagreement statements based on best practices for the method (Markowitz, 2021). The data were well-suited for PCA using language patterns (Kaiser-Meyer-Olkin Measure of Sampling Adequacy = 0.518, Bartlett’s Test of Sphericity = χ^2^(120) = 276.63, *p* < .001), and 16 unigrams (single words) were retained. Items (words) were retained if the contained loadings were greater than |.20| after varimax rotation.

Two reliable components were extracted from the Meaning Extraction Method after considering a scree plot, the amount of variance explained by components, and overall thematic interpretability. The two components, addressing topics PolitiFact emphasized that *The Washington Post* did not, accounted for 22.86% of the variance and are shown below. Component 1 reflects the theme of President Trump proclaiming then-candidate Joe Biden would have illegal plans as president and would also help illegal immigrants. For example, Trump suggested “*Joe Biden and Kamala Harris’ government-run health care plan… could lead to hospitals being closed, put Medicare coverage at risk, and give benefits to illegal immigrants*,” which was fact-checked by PolitiFact but did not appear on *The Washington Post*’s Fact Checker. Component 2 discussed the integrity of the 2020 US presidential election *(*“*Phony” ballots “were printed without my name on it.*”), checked by PolitiFact but not *The Washington Post*. Taken together, from this analysis, selection disagreements were systematically about two main topics, even though the topics themselves were widely covered in the news.

*Results of the Principal Component Analysis*

| Component 1:  Biden's illegality | | Component 2:  Election integrity | |
| --- | --- | --- | --- |
| λ = 2.15 | 13.46% | λ = 1.50 | 9.40% |
| Word | Loading | Word | Loading |
| Joe | 0.898 | ballot | 0.806 |
| Biden | 0.861 | election | 0.746 |
| plan | 0.510 | win | 0.281 |
| illegal | 0.466 | law | 0.238 |

# **Expanded Investigation into PolitiFact and *The Washington Post* Discrepancy**

In the article’s main text, we report that the verbatim check for *true* and *mostly true* statements found that only 1 out of 64 statements (1.6%) was given a false and misleading rating and included in *The Washington Post* selection, when PolitiFact tagged it as *mostly true*. The statement in question is: “In 2016, an officer was assaulted in America on an average of every 10 minutes.” In their assessment of veracity, PolitiFact stated: “Trump's statement is accurate but requires additional context. We rate it Mostly True.” In their assessment of veracity, *The Washington Post* stated: “Trump is skating a fine line. His math checks out, but the context how he is presenting "assault" is misleading.” Both sources suggest more context is needed, but they provided different veracity ratings. We believe this statement is more aligned with “misleading” than “false” for *The Washington Post*, which would also fit the “Mostly True” description by PolitiFact.

# **References**

Chung, C. K., & Pennebaker, J. W. (2008). Revealing dimensions of thinking in open-ended self-descriptions: An automated meaning extraction method for natural language. *Journal of Research in Personality*, *42*(1), 96–132. https://doi.org/10.1016/J.JRP.2007.04.006

Markowitz, D. M. (2021). The meaning extraction method: An approach to evaluate content patterns from large-scale language data. *Frontiers in Communication*, *6*, 13. https://doi.org/10.3389/fcomm.2021.588823
